# Penetrating Sacral Injury with a Wooden Foreign Body

**DOI:** 10.1155/2018/1630864

**Published:** 2018-04-19

**Authors:** Arash Fattahi, Amin Jahanbakhshi, Ardeshir Shahivand, Alireza Dastmalchi

**Affiliations:** Neurosurgery, Iran University of Medical Sciences, Tehran, Iran

## Abstract

Spinal penetrating trauma has a complex neurosurgical management. This study presents a 55-year-old male admitted in our center with a 1 × 1 centimeter laceration on the sacral area skin due to a wooden penetrating object. The computed tomography (CT) of the spine revealed a hypodense penetrating object that was introduced from the posterior aspect of the sacrum into dural space and then stopped in the S1 vertebral body. We performed a laminectomy of intact superior portion of the S1 lamina and released the wood from the surrounding bone, and finally, we pulled it out.

## 1. Introduction

Spinal penetrating trauma has a complex neurosurgical management. The complications such as meningitis, osteomyelitis, wound infection, and neurological deficits increase the complexity of treating these patients [1]. Also, their surgical management is accompanied with some possible complications such as cerebrospinal fluid (CSF) leakage. A true diagnosis and fine management can improve the overall outcome of these patients. After searching databases all over the world, we could not find any report of penetrating wood in the sacral area.

## 2. Case Report

**Figure 1:**
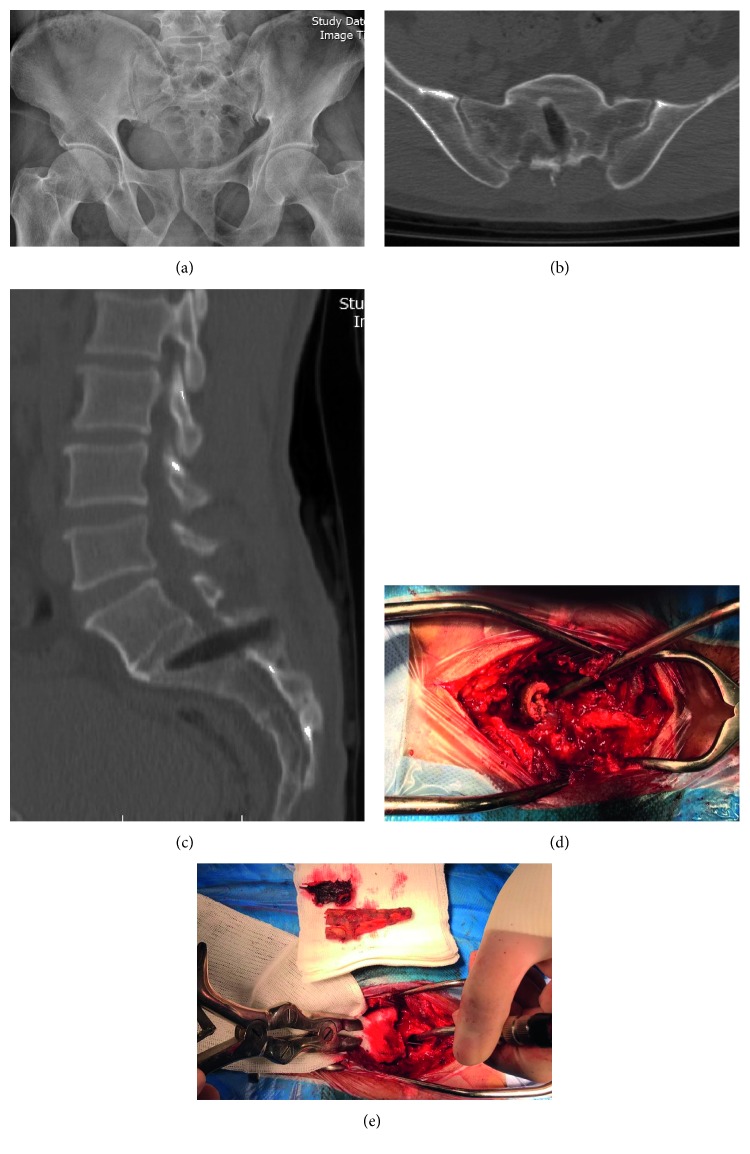
A pelvic X-ray shows sacral bone defect (a). The axial (b) and sagittal (c) CT scan of lumbosacral area shows a hypodense penetrating object that was introduced from the posterior aspect of the sacrum and then stopped in the S1 vertebral body. Also, intraoperative illustration can be observed before (d) and after (e) wood removal.

This study presents a 55-year-old male admitted in our center with a 1 × 1 centimeter laceration on the sacral area skin, 12 cm above the anus. He had fallen from a mulberry tree and had removed the sunken tree branch. He presented no neurological deficit or fever and had a normal sphincter function. The laceration was dirty with slow CSF leakage. The pelvic X-ray showed an area of defect in the sacral region (Figure 1(a)). The computed tomography (CT) of the spine revealed a hypodense penetrating object that was introduced from the posterior aspect of the sacrum into the dural space and then stopped in the S1 vertebral body, and the object did not penetrate the anterior cortex of the vertebral body (Figures 1(b) and 1(c)).Because of the possibility of pelvic organ injury in cases of penetrating sacral injury, we consulted with an expert general surgeon. Based on normal findings on physical exam and pelvic CT scanning, our consultant surgeon assured us. Thereafter, the patient was transferred to the operating room. Under general anesthesia, the patient was operated in prone position. Muscle dissection revealed a wooden foreign body on the sacral lamina that passed into the S1 body (Figure 1(d)). First, a laminectomy of an intact superior portion of the S1 lamina was performed and the wood was released from the surrounding bone, and finally, it was pulled out. Before the wood was removed, the dural sac was mildly compressed above the level continuously, to prevent CSF from filling our surgical field. Also, a portion of the patient's clothes was removed from the vertebral body (Figure 1(e)). The bone defect was massively irrigated, and the defect of the dura was repaired with Prolene suture separately in a watertight manner. Thereafter, the Valsalva maneuver was performed and no CSF leakage was observed. Finally, the surgical wound was closed in a layer-by-layer manner. The patient was under close observation for 3 days and then discharged without any deficit or complication. The patient was visited 2 weeks later and it was observed that his wound had healed. Unfortunately, as a result of the different nationality of the patient (Afghanistan), we did not see him again, but we were informed by phone that he was still doing fine, 8 months after the procedure.

## 3. Discussion

Only very few reports were found on spinal penetrating trauma with wooden objects; hence, it is extremely rare [2]. All over the world, we were unable to find any report on penetrating wood in the sacral area. Although the first sacral root injury is possible, the only sphincter disturbance is more prevalent in this area and other neurological deficits are less common. In these situations, performing an accurate physical examination can reveal any preoperative deficits and target planning.

As a result of the structural components of wood and its large air content, it is difficult to see it on imaging protocols. Wooden objects are usually missed on plain radiographs. On the CT scan, wood is a hypodense mass and, sometimes, it could be confused as air. However, performing a CT scan can identify sites of foreign body and its trajectory [3]. Imokawa et al. suggested magnetic resonance imaging (MRI) as an adjunctive tool for the diagnosis of wooden objects [4]. The careful preoperative imaging can help us for accurate surgical planning to remove all wooden foreign body portions. MRI was not performed because our patient had CSF leakage from the dirty wound, and it was necessary to treat him as soon as possible. On the other hand, a foreign body was seen clearly on the CT scan.

As a result of their organic nature, wooden objects are sources of infection and all wooden foreign bodies should be totally removed from the wound carefully [2, 5]. The surgical management of these patients should include high volume irrigation. Meticulous repairing of any tearing of the dura is the rule to prevent subsequent meningitis. The fascia should be repaired to prevent subsequent leakage. We routinely do not prescribe widespread antibiotics for these patients. Anyway, a true diagnosis and precise treatment can forecast a good final prognosis for these patients.

## 4. Conclusion

This study presents an extremely rare case of sacral penetrating injury with a wooden object. The surgical removal of the foreign body is recommended as soon as possible. In these cases, meticulous repairing of torn dura matter and fascia must be the cornerstone of our surgical strategy.
